# Establishment of Hairy Root Cultures of *Rhaponticum carthamoides* (Willd.) Iljin for the Production of Biomass and Caffeic Acid Derivatives

**DOI:** 10.1155/2015/181098

**Published:** 2015-02-24

**Authors:** Ewa Skała, Agnieszka Kicel, Monika A. Olszewska, Anna K. Kiss, Halina Wysokińska

**Affiliations:** ^1^Department of Biology and Pharmaceutical Botany, Medical University of Łódź, Muszyńskiego 1, 90-151 Łódź, Poland; ^2^Department of Pharmacognosy, Medical University of Łódź, Muszyńskiego 1, 90-151 Łódź, Poland; ^3^Department of Pharmacognosy and Molecular Basis of Phytotherapy, Medical University of Warsaw, Banacha 1, 02-097 Warsaw, Poland

## Abstract

The aim of the study was to obtain transformed roots of *Rhaponticum carthamoides* and evaluate their phytochemical profile. Hairy roots were induced from leaf explants by the transformation of *Agrobacterium rhizogenes* strains A4 and ATCC 15834. The best response (43%) was achieved by infection with A4 strain. The effects of different liquid media (WPM, B5, SH) with full and half-strength concentrations of macro- and micronutrients on biomass accumulation of the best grown hairy root line (RC3) at two different lighting conditions (light or dark) were investigated. The highest biomass (93 g L^−1^ of the fresh weight after 35 days) was obtained in WPM medium under periodic light. UPLC-PDA-ESI-MS^3^ and HPLC-PDA analyses of 80% aqueous methanol extracts from the obtained hairy roots revealed the presence of eleven caffeoylquinic acids and their derivatives and five flavonoid glycosides. The production of caffeoylquinic acids and their derivatives was elevated in hairy roots grown in the light. Only light-grown hairy roots demonstrated the capability for the biosynthesis of such flavonoid glycosides as quercetagetin, quercetin, luteolin, and patuletin hexosides. Chlorogenic acid, 3,5-di-*O*-caffeoylquinic acid and a tentatively identified tricaffeoylquinic acid derivative were detected as the major compounds present in the transformed roots.

## 1. Introduction

*Rhaponticum carthamoides* (Willd.) Iljin, a member of the Asteraceae family, is a perennial, herbaceous species naturally growing in the mountains of South Siberia, Middle Asia, and Mongolia. It is commonly known as “maral root” or Russian leuzea and has been used for centuries in traditional Siberian medicine as a stimulant, mostly in the case of overstrain and weakness after illness [1]. The root and rhizome extracts of* R. carthamoides* possess a wide range of biological activities, including adaptogenic, antioxidant, cardioprotective, immunomodulatory, antihyperlipidemic, antihyperglycemic, and antimicrobial effects [1]. These pharmacological properties are attributed to the presence of a variety of secondary metabolites including triterpenoids, polyacetylenes, sesquiterpene lactones, phenolic acids, flavonoids, and ecdysteroids with 20-hydroxyecdysone as the principal component [1].

The medicinal importance and endangered status of* R. carthamoides*, have resulted in its cultivation worldwide, including Central and Eastern Europe. However, 3-4 years are required to obtain plant roots with a satisfactory content of the pharmacologically active compounds by field cultivation. In addition, it is inefficient to harvest the roots from field-grown plants as these results in the loss of the mother plant. It would be desirable to develop an effective biotechnological method for the production of suitable plant material in a shorter time period, regardless of seasonal and climatic conditions. One approach could be the use of hairy root cultures transformed by* Agrobacterium rhizogenes*. This type of* in vitro* culture has gained considerable attention because of their fast growth in media without growth regulators, their genetic and biochemical stability and their ability to biosynthesise selected secondary metabolites at levels comparable to, or even higher than, those found in roots of intact plants [2, 3]. A previous work [4] reports that transformed root cultures of* R. carthamoides* were found to be ineffective for ecdysone production, but no information is available regarding polyphenol accumulation and no optimization data is given for biomass production by the culture.

The aim of this study was the establishment of hairy roots of* R. carthamoides* and the phytochemical profiling of their polyphenolic constituents. Two* A. rhizogenes* strains (A4 and ATCC 15834) and leaf explants were used for hairy root induction. The effect of different liquid nutrient media (WPM, B5, SH and 1/2 WPM, 1/2 B5, 1/2 SH) and culture conditions (light or dark) on hairy root growth, in terms of fresh and dry biomass accumulation, were also investigated. The incorporation of T-DNA genes into the plant genome was demonstrated by PCR analysis.

Furthermore, the hairy root cultures exhibiting the highest biomass productivity and roots of the soil-cultivated plants of* R. carthamoides* were characterized to their main phytochemical markers, including caffeoylquinic acids, flavonoid glycosides, and 20-hydroxyecdysone. The comprehensive qualitative and quantitative phytochemical profiling of the plant samples was performed by UPLC-PDA-ESI-MS^3^ and HPLC-PDA methods.

## 2. Material and Methods

### 2.1. Plant Material and Bacterial Strains

The leaves of four-week-old* in vitro* shoots of* R. carthamoides* (derived from seeds obtained from the Medicinal Plant Garden of the Department of Pharmacognosy, Medical University of Łódź, Poland) cultured on Murashige and Skoog (MS) agar (0.7%) medium [5] containing 0.1 mg L^−1^ indole-3-aceticacid and 0.2 mg L^−1^ benzyladenine were used as a explants for* Agrobacterium rhizogenes*-mediated transformation. Botanical identity of plants was confirmed by E. Skała according to Flora of China (http://www.efloras.org/). The voucher specimen was deposited at the Department of Biology and Pharmaceutical Botany, Medical University of Łódź (Poland).

Two agropine-type strains of* Agrobacterium rhizogenes* (A4 and ATCC 15834) were used for hairy root induction. The bacteria were grown for 48 h on YEB solid (1.5%) medium [6], at 26°C in the dark.

### 2.2. Induction and Establishment of Hairy Root Culture

The leaf explants were wounded with a sterile needle immersed in the bacterial culture. Inoculation was carried out in the middle part of the petiole or at the basal part of the leaf lamina. Control explants were wounded identically with sterile needle without bacteria. Infected and control explants were placed on hormone-free MS agar (0.7%) medium with or without acetosyringone (AcS) (200 *μ*M) and incubated in the dark for 5 weeks. The experiment was repeated three times; 25–35 explants were used for each treatment: type of bacterial strain/site of infection/medium with or without AcS.

Five weeks after initial inoculation, the transformation frequency (the percentage of explants forming roots after infection with* A. rhizogenes* with respect to total number of infected explants), the number of roots per responding explant, and the root length were determined (Table 1).

### 2.3. Liquid Culture of Hairy Roots

**Figure 1:**
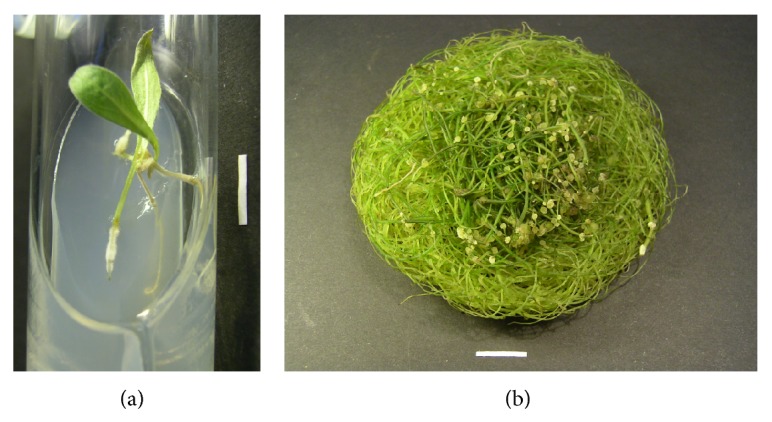
The hairy roots of* R. carthamoides*. (a) Induction of the hairy roots of the leaves after 4 weeks on MS agar (0.7%) medium. (b) Light-grown hairy roots cultured in 300 mL Erlenmeyer flasks containing 80 mL phytohormone-free WPM liquid medium after 35 days (*bar* = 1 cm).

Adventitious roots (1-2 cm long) (Figure 1(a)) were excised from explants and transferred individually into 100 mL Erlenmeyer flasks containing 20 mL half-strength Gamborg (1/2 B5) liquid medium [7] without growth regulators and supplemented with 500 mg L^−1^ ampicillin for the elimination of the bacteria. The cultures were maintained in the dark, on a rotary shaker at 80 rpm. After several subcultures of 7 days each, the concentration of ampicillin was reduced to 300 mg L^−1^. After four successive subcultures, the antibiotic was eliminated from the medium and eight axenic root lines were obtained (RC1–RC8). Among them, line RC3, showed the fastest growth and produced more lateral roots than the other seven lines. Therefore, this line was chosen for further experiments.

### 2.4. Culture of* R. carthamoides* Hairy Roots in Different Media

Six different liquid media were tested for their effect on root biomass production: Schenk and Hildebrandt (SH) [8], Woody Plant (WPM) [9], and Gamborg (B5) with full and half-strength macro- and microsalt concentration (1/2 SH, 1/2 WPM, 1/2 B5). The media contained 3% sucrose. The experiments were carried out in 300 mL Erlenmeyer flasks containing 80 mL of the liquid medium. The flasks were maintained on a rotary shaker at 80 rpm, in the darkness or under a 16/8 h light/dark photoperiod; PPFD of 40 *μ*mol m^−2^ s^−1^ using cool-white fluorescent lamp. Subcultures were carried out every 5 weeks by transferring 0.4–0.6 g of fresh root biomass into fresh medium. Measurement of biomass accumulation was calculated as both fresh (FW) and dry (DW) weights after 5 weeks of culture. The morphology of hairy roots (colour, thickness, and intensity of branches) was also evaluated. Three flasks from three successive subcultures (15–17 passages) were used for each medium type and culture conditions (darkness/photoperiod). The thickness of the hairy roots was estimated with the Motic Images Plus 2.0 ML computer application (2006) connected to a light microscope (Delta Optical Genetic Pro) (Poland). The hairy roots were found to be stable in terms of increase in root biomass and their morphology.

### 2.5. DNA Isolation and PCR Analysis

Genomic DNA was extracted from fresh hairy roots (RC3 line) and nontransformed shoots of* R. carthamoides* using NucleoSpin Plant II Kit (Macherey-Nagel, Germany). The plant materials (200 mg) were powdered in liquid nitrogen. Each DNA sample was used as a template for PCR analysis to determine the presence of the* rol*A,* rol*B,* rol*C,* rol*D,* aux*1, and* aux*2 genes in the T-DNA. In order to confirm that the hairy roots were correctly transformed, PCR was performed using* vir*G gene. The sequence of primers used to amplify the genes is given in Table 2. The Ri plasmid was isolated from 24 h cultures from* Agrobacterium rhizogenes*, strain A4 (OD_600_ = 0.5) using Plasmid Mini AX Kit (A&A Biotechnology, Poland).

PCR amplification was performed in a 25 *μ*L volume containing 5 *μ*L DNA solution, 5 *μ*L of each primer (forward and reverse), 2.5 *μ*L 10x* Taq*Nova reaction buffer (Blirt, Poland), 1.25 *μ*L MgCl_2_ (2.5 mM), 2 *μ*L dNTP mix (0.2 mM) (Blirt, Poland), and 0.6 *μ*L 2 U/*μ*L* Taq*Nova DNA polymerase (Blirt, Poland). The PCR programme comprised 36 cycles in which first denaturation was carried out at 95°C for 2.30 min, segment denaturation at 95°C for 0.30 min, annealing at 55°C for 0.30 min, extension at 72°C for 1.10 min, and final extension for 3 min at 72°C. The PCR products were analyzed with a 100 bp DNA ladder on 1.2% agarose gel (Bioline, UK) for 1 h at a constant voltage of 90 V in TBE buffer. The gel was stained with ethidium bromide, visualized under UV light, and photographed using DNR Bio-Imaging System MiniBIS Pro (Israel).

### 2.6. Phytochemical Analysis

#### 2.6.1. Chemicals and Standards

The standards of HPLC-grade purity (≥96%) such as chlorogenic acid (5-*O*-caffeoylquinic acid, CHA), cynarin (1,3-di-*O*-caffeoylquinic acid, CA), and isoquercitrin (quercetin 3-*O*-*β*-d-glucopyranoside, IQ) were purchased from Fluka (Germany) and 20-hydroxyecdysone (EC) from Sigma-Aldrich (Germany/USA). HPLC grade solvents, acetonitrile, orthophosphoric acid, and redistilled water were obtained from POCH (Poland) and Merck (Germany).

#### 2.6.2. Extraction Procedure

An accurately weighed sample of lyophilized and powdered plant material was first extracted with* n*-hexane. The samples were 600 mg for the 35-day-old hairy roots cultured in WPM medium in the light (HR-L) or in the dark (HR-D) and 300 mg for the roots of soil-grown 3-year-old plants (SR). After filtration, the* n*-hexane extract was discarded. The defatted sample was sonicated for 15 min with 80% (v/v) aqueous methanol (25 mL) at 35°C using an ultrasonic bath and then twice with 10 mL of the same solvent for 15 min. The combined extracts were diluted with methanol to 50 mL, filtered through a PTFE syringe filter (25 mm, 0.2 *μ*m, AlChem, Czech Republic) and the filtrate was directly injected into the HPLC or UPLC system.

#### 2.6.3. Qualitative UPLC-PDA-ESI-MS^3^ Analysis

The UPLC-PDA-ESI-MS^3^ analysis was performed using an UPLC-3000 RS system (Dionex, Germany) equipped with a dual low-pressure gradient pump, an autosampler, a column compartment, a diode array detector, and an AmaZon SL ion trap mass spectrometer with an ESI interface (Bruker Daltonik, Germany). The samples were separated on a Kinetex XB-C18 column (1.7 *μ*m, 150 × 2.1 mm i.d., Phenomenex, USA). The mobile phase consisted of solvent A (0.1% aqueous solution of formic acid, v/v) and solvent B (acetonitrile with 0.1% formic acid, v/v) with an elution profile as follows: 0−45 min 6−26% B (v/v), 45–55 min 26–95% B, 55–63 min 95% B, and 63–70 min 95–6% B. The flow rate was 0.3 mL min^−1^, the column temperature was maintained at 25°C. The UV-Vis spectra were recorded over the range 200−600 nm, and chromatograms were acquired at 245, 325, and 350 nm. The LC eluate was introduced directly into the ESI interface without splitting. The nebulizer pressure was 40 psi; dry gas flow 9 L min^−1^; dry temperature 300°C; and capillary voltage 4.5 kV. The analysis was carried out using a scan from* m/z* 200 to 2200. The compounds were analyzed in a negative ion mode.

#### 2.6.4. Quantitative HPLC-PDA Analysis

The HPLC-PDA analysis was performed on the Waters 600E Multisolvent Delivery System (Waters, USA) with a PDA detector (Waters 2998) working in the range of 220–450 nm, a model 7725 sample injection value (Rheodyne, CA, USA), a 5 *μ*L injection loop, and a LC workstation equipped with Waters Empower 2 software for data collection and acquisition. The analytical column was a C18 Ascentis Express (2.7 *μ*m, 75 mm × 4.6 mm i.d.; Supelco, PA, USA), guarded by a C18 Ascentis C18 Supelguard column (3 *μ*m, 20 mm × 4 mm i.d.; Supelco). The mobile phase consisted of solvent A (0.5% aqueous solution of orthophosphoric acid, w/v) and solvent B (acetonitrile) with an elution profile as follows: 0-1 min 5% B (v/v), 1–16 min 5–30% B, 16-17 min 30–50%, 17–19 min 50% B, 19-20 min 50–5% B, and 20–25 min 5% B (equilibration). The flow rate was 1.4 mL min^−1^ and the column temperature was maintained at 30°C. The phenolic compounds were identified and classified into three groups based on their UV-Vis spectra, retention times, and the qualitative results obtained from UPLC-PDA-ESI-MS^3^ (the accurate mass and the MS fragmentation patterns). The detection wavelength was set at 245 nm for 20-hydroxyecdysone, 325 nm for caffeic acid derivatives including caffeoylquinic acids, and 350 nm for the flavonoid glycosides. Four external standards were used for calibration including chlorogenic acid (CHA), cynarin (CA), 20-hydroxyecdysone (EC), and isoquercitrin (IQ). The calibration equations were constructed using seven concentration levels of each analyte within the range of approximately 2.4–240 *μ*g mL^−1^ for CHA, 1.0–100 *μ*g mL^−1^ for CA, 1.0–107 *μ*g mL^−1^ for EC, and 1.0–103 *μ*g  mL^−1^ for IQ. The tentatively identified peaks were quantified as equivalents of the following standards: chlorogenic acid isomers as CHA, dicaffeoylquinic acid isomers, tricaffeoylquinic acid and its derivative as CA, 20-hydroxyecdysone as EC, and the flavonoid monoglycosides as IQ.

### 2.7. Statistical Analysis

The statistics (calculation of RSD and SE, one-way analysis of variance, significance tests, and linearity studies) were performed using the software Statistica 10.0PL for Windows (StatSoft Inc., Poland).

## 3. Results and Discussion

### 3.1. Induction of Hairy Roots

The first adventitious roots were visible 2-3 weeks after inoculation. No roots were observed in noninfected control explants. The highest frequency of hairy root induction was achieved on explants infected with strain A4 and cultured on MS medium supplemented with AcS. It was 43.3% when leaf explants were wounded at the lamina base and 37.3% after infection at the middle part of the petiole. The differences were not statistically significant (*P* ≥ 0.05) (Table 1). It has been well documented that bacterial strains differ in their virulence and the choice of the appropriate strain is an important factor for successful transformation [10, 11]. After infection with strain ATCC 15834, 18.3% of lamina and 22.7% of petiole explants responded by producing roots after 5 weeks of culture on MS medium containing AcS (200 *μ*M) (Table 1). The site of infection also did not have any significant effect on frequency of hairy root induction.

Acetosyringone (AcS) has been reported to induce the expression of* vir* genes and thus affect* Agrobacterium*-mediated transformation [12]. Therefore, it was added to* R. carthamoides* root induction medium at the concentration of 200 *μ*M, a concentration which was found to have a positive effect on the transformation frequency of* Picrorhiza kurroa* [13]. It was found that the addition of 200 *μ*M AcS to MS medium for* R. carthamoides* root initiation increased the number of explants producing roots, although the effect was not statistically significant at *P* = 0.05 (Table 1). The relatively low differences in root formation between treatments given with and without AcS suggest that this compound is not a key factor in the transformation of* R. carthamoides*, which may be due to the high level of phenolic compounds present in the leaf explants.

### 3.2. Growth of Hairy Roots in Different Liquid Media

**Figure 2:**
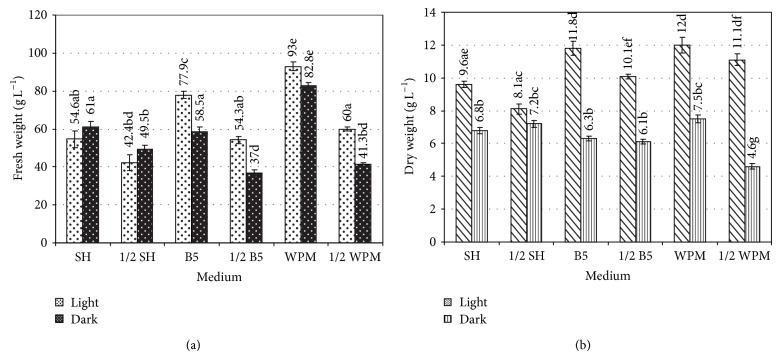
Comparative growth of* R. carthamoides* transformed roots after 35 days in liquid medium in the light (photoperiod: 16 h of light: 40 *μ*mol m^−2^ s^−1^) and in the dark. (a) Fresh weight (g) of harvested biomass per liter. (b) Dry weight (g) of harvested biomass per liter. The values are the mean ± SE of three successive subcultures (15–17). Means followed by the same letter at the columns (individually to fresh and dry weight) are not significantly different at the level of *P* ≥ 0.05 (the Mann-Whitney *U* test).

The hairy roots (RC3 line) grown in full strength media possessed greater biomass than roots cultured in half-strength media (Figures 2(a) and 2(b)). This was similar to hairy root cultures of* Levisticum officinale* grown in B5 medium, which showed a greater increase in biomass (350 g L^−1^ FW and 10 g L^−1^ DW) than roots grown in 1/2 B5 medium (200 g L^−1^ FW and 7 g L^−1^ DW) [14]. In the present study, the highest accumulation of hairy root biomass was achieved in WPM medium with a full concentration of nutrients. After 35 days, the fresh weight of hairy roots was 93 g L^−1^ grown in photoperiod and 82.8 g L^−1^ for roots cultured in the dark (Figure 2(a)). The values for dry weights were 12.0 g L^−1^ and 7.5 g L^−1^, respectively (Figure 2(b)). The roots were thin with an average root diameter of 0.5 mm and had long and numerous branches (Figure 1(b)). The WPM medium was also the best for the growth of transformed roots of other plant species like as* Trigonella foenum-graecum* [15] or* Dracocephalum moldavica* [16]. Of the tested media, SH, B5, and WPM with half-strength macro- and microsalt concentration were found to induce the lowest level of* R. carthamoides* root biomass in terms of both fresh and dry weights (Figures 2(a) and 2(b)). The transformed roots maintained in SH and B5 media with full and half-strength content of macro- and micronutrients were thick with an average root diameter of 0.9–1.5 mm and had short and small branches. Generally, exposure to light increased the growth of hairy roots of* R. carthamoides*, except for the roots cultured in SH and 1/2 SH media. In these media fresh weights of the roots grown under photoperiod were lower than those achieved under darkness. However, the differences were not statistically significant at *P* = 0.05. The hairy roots were found to be stable in terms of increase in root biomass and their morphology. The physical culture conditions affected also the morphology of* R. carthamoides* hairy roots; that is, roots cultured in the photoperiod were green (Figure 1(b)). Greening was absent in the dark-grown roots which were beige. The previous report results showed that exposure of hairy root cultures to light can induce greening due to enhanced chlorophyll biosynthesis [17, 18].

### 3.3. PCR Analysis

**Figure 3:**
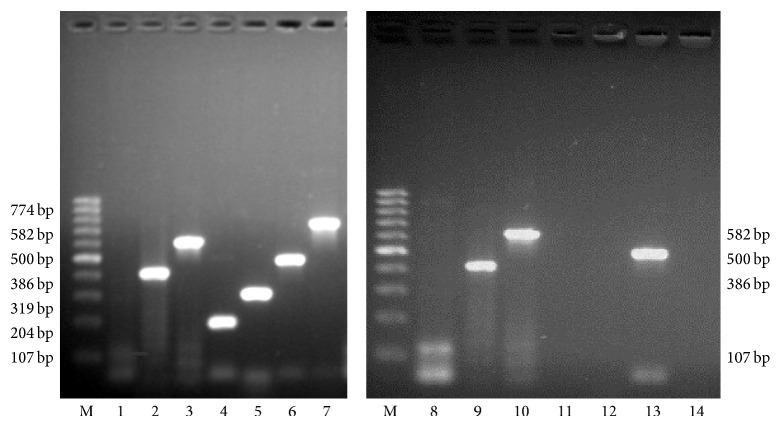
PCR analysis of genomic DNA isolated from* Rhaponticum carthamoides* hairy roots.* Lanes*: M: molecular weight marker (100 bp DNA ladder); 1–7 positive control (plasmid DNA from* A. rhizogenes* strain A4), DNA showing* rol*A (107 bp),* rol*B (386 bp),* rol*C (582 bp),* rol*D (204 bp),* vir*G (319 bp),* aux*1 (500 bp), and* aux*2 (774 bp) genes; 8–14 genomic DNA of hairy roots (RC3 line).

The genetic transformation of the* R. carthamoides* hairy roots was confirmed by PCR. Using specific PCR primers four amplified bands of expected size 107 bp, 386 bp, 582 bp, and 500 bp corresponding to the* rol*A,* rol*B,* rol*C, and* aux*1 genes, respectively, appeared in the hairy root line RC3 (Figure 3, lanes 8, 9, 10, and 13, resp.) but not in the nontransformed shoots used as a negative control. PCR analysis was carried out using primers specific to* vir*G to confirm that hairy roots were not contaminated with* A. rhizogenes* (Figure 3, lane 12).

The results of the PCR analysis revealed the insertion of both T_L_-DNA (the presence of A, B, C* rol* gene fragments) and T_R_-DNA (the presence of the* aux*1 gene fragment) into the genome of* R. carthamoides* hairy roots.

### 3.4. Identification of Polyphenols and 20-Hydroxyecdysone in Hairy Roots and Roots of Soil-Grown Plants by UPLC-PDA-ESI-MS^3^

The UPLC-PDA-ESI-MS^3^ studies of 80% aqueous methanol extracts of HR-L and HR-D hairy roots cultured in liquid medium WPM and roots of soil-grown 3-year-old plants (SR) of* R. carthamoides* resulted in full or partial identification of 17 compounds comprising caffeoylquinic acids and their derivatives, 20-hydroxyecdysone and flavonoids (Table 3). The detected compounds were identified by comparing their retention times, UV-Vis spectra, and fragmentation patterns in MS spectra with those of the reference compounds and the literature data [19–22].

The UPLC-PDA study showed that the caffeoylquinic acids and their derivatives constitute the major phenolic class occurring in both the hairy roots and the roots of soil-grown plants. These compounds (peaks** 1**–**4** and** 11**–**15**), based on their parent ions in the negative mode ([M−H]^−^ at* m/z* 353, 515, and 677), were classified into three groups: mono-, di-, and tricaffeoyl esters of quinic acid. The monocaffeoylquinic acids (compounds** 1**–**3**) were located on the chromatograms by their parent deprotonated ions [M−H]^−^ at* m/z* 353. Their preliminary identification was facilitated by analysis of structure-diagnostic hierarchical keys proposed by Clifford et al. [19]. Compounds** 1** and** 2**, exhibiting the MS^2^ base ions at* m/z* 191 and the secondary ions at* m/z* 179 of the intensity of 43% and 4%, respectively, were identified as NCHA (neochlorogenic acid, 3-*O*-caffeoylquinic acid) and CHA (chlorogenic acid, 5-*O*-caffeoylquinic acid), respectively. The third isomer (compound** 3**), due to its MS^2^ base ion observed at* m/z* 173, was assigned as CCHA (cryptochlorogenic acid, 4-*O*-caffeoylquinic acid). Finally, the unequivocal identification of compounds** 1**–**3** was confirmed by comparison of their retention time and MS data with the commercially available standard of CHA as well as with the qualitative standards of NCHA and CCHA prepared in our laboratory according to Clifford et al. [23].

The further five compounds (**4** and** 11**–**14**) (Table 3), eluting after chlorogenic acid, were classified as dicaffeoylquinic acids, all of which exhibited the UV-Vis absorption maxima at 325 or 328 nm and whose deprotonated molecular ions were found at* m/z* 515. In the MS^2^ spectra, these compounds gave base peaks at* m/z* 353 ([M-H-caffeoyl]^−^) and the secondary ions at* m/z* 335 or 191 with varying intensities (10–30% of the base peak). Additionally, at the MS^3^ level, the base peak at* m/z* 173 was characteristic of the isomers with a caffeoyl moiety substituted at position C-4 of quinic acid, whereas other substitutions gave base peaks at* m/z* 191. A comparison of the elution order and the fragmentation patterns of the product ions described above to those reported in the literature for dicaffeoylquinic acids [21, 24] suggests that the detected isomers** 4** and** 11**–**14** were 1,3-; 3,4-; 3,5-; 1,5-; and 4,5-*O*-dicaffeoylquinic acids, respectively. Finally, compound** 4** was compared with the commercial standard of cynarin (1,3-di*-O-*caffeoylquinic acid).

A search for tricaffeoylquinic acids with the deprotonated molecular ion at* m/z* 677 and typical UV-Vis absorption maxima at 325 nm resulted in the identification of one chromatographic peak. On the basis of its MS^2^ base peak at* m/z* 497 and the secondary ion at* m/z* 515 (20% of base peak intensity), which yielded an MS^3^ base peak at* m/z* 353, the compound** 15** was identified as 1,4,5-tri-*O*-caffeoylquinic acid, according to the literature data [20, 21].

As shown in Table 3, there are two further compounds with UV-Vis spectra typical of caffeic acid derivatives with absorption maxima at 327–329 nm. These compounds, eluting after compound** 15**, both with the deprotonated molecular ions at* m/z* 793, produced similar MS^2^ and MS^3^ base peaks at* m/z* 631 ([M-H-caffeoyl]^−^) and 469 ([M-caffeoyl-caffeoyl−H]^−^), respectively. In the MS^3^ spectra, neutral losses of 116 mass units were also observed forming the fragment ions at* m/z* 353 characteristic of monocaffeoylquinic acid. Based on these results, the detected compounds** 16** and** 17** might be only tentatively identified as two isomers of tricaffeoylquinic acid substituted with an unidentified group. Due to the lack of suitable reference standards and literature data, the complete identification of these compounds needs isolation and full spectral characterization.

The analyzed extract of soil-grown roots only gave one peak with a UV-Vis spectrum demonstrating absorption maxima at 247 nm, which is typical of ecdysones. The MS spectrum revealed its molecular mass to be 480 amu based on [M+HCOO]^−^ ion at* m/z* 525 and [M−H]^−^ at* m/z* 479. A comparison of these spectral data with those obtained from the authentic standard allowed to confirm the identification of 20-hydroxyecdysone (compound** 8**) (Table 3).

The other group of compounds exhibits the UV-Vis spectra characteristic of flavonoids with two absorption maxima, first at 250–260 nm and second at 350–370 nm. All compounds** 5**–**7** and** 9**-**10** were identified as flavonoid hexosides due to neutral losses of 162 mass units in their MS^2^ spectra. Flavonoid** 5** was assigned as a quercetagetin hexoside, since the MS study of its deprotonated molecular ion (*m/z* 479) provided a characteristic product ion at* m/z* 317 in the negative mode MS of quercetagetin aglycone [22]. Likewise, compound** 10** was tentatively identified as patuletin hexoside by comparing its UV-Vis spectrum and fragmentation pattern of the aglycone moiety in MS^3^ spectrum with the literature [22]. Compounds** 6** and** 7** had identical MS profiles: fragmentation of deprotonated ion [M−H]^−^ at* m/z* 463 yielded the base ion at* m/z* 301 in MS^2^ which corresponded to either of the two characteristic aglycones of* R. carthamoides*, quercetin, or 6-hydroxykaempferol [25–27]. According to the literature data, the MS spectra of quercetin and 6-hydroxykaempferol reveal the presence of characteristic fragment ions at* m/z* 151 and* m/z* 167, respectively [22]. Thus, according to the observed MS^3^ fragmentation (Table 3), the aglycones of compounds** 6** and** 7** were assigned as quercetin. The MS^2^ spectrum of compound** 9** revealed an ion of aglycone moiety at* m/z* 285, which could suggest the presence of luteolin or kaempferol. The comparison of the UV-Vis spectrum of** 9** and the fragmentation pattern of its aglycone in MS^3^ spectrum with the literature [22, 28, 29] indicated the presence of luteolin.

### 3.5. Quantitative HPLC-PDA Analysis

**Figure 4:**
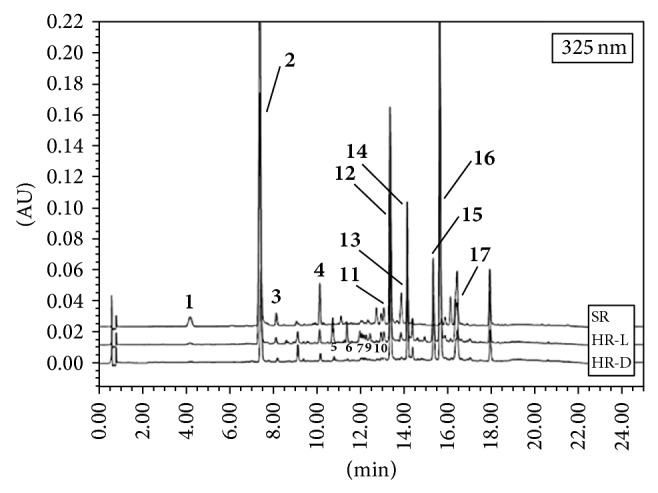
Representative HPLC-UV (325 nm) chromatograms of* R. carthamoides* root extracts. Sample and peak codes are given in Table 4.

The contents of mono-, di-, and tricaffeoylquinic acids and their derivatives and flavonoid glycosides in HR-L an HR-D hairy roots selected from optimum medium (WPM) were determined by HPLC-PDA analysis and compared with the roots of 3-year-old nontransformed plants of* R. carthamoides* grown in the soil (Table 4 and Figure 4). The growing interest of caffeoylquinic acids and their derivatives is based on their diverse biological activities which include anti-inflammatory, analgesic, antipyretic, and anticarcinogenic effects [30, 31]. Caffeoylquinic acids are free radical and metal scavengers and have been shown to modulate the gene expression of antioxidant enzymes [32]. Also, they have neuroprotective, neurotrophic [33], and hepatoprotective activity [34].

The results indicate that the total concentration of caffeoylquinic acids and their derivatives (calculated as the sum of compounds** 1**–**4** and** 11**–**17**) (Table 4) was about 2-times higher in hairy roots cultured in photoperiod (HR-L) (19.08 mg g^−1^ DW) than that found in dark-grown hairy roots (HR-D) (11.45 mg g^−1^ DW) (Table 4). The mean individual caffeic acid derivative content followed a similar pattern. It suggests a regulation response to light of the phenylpropanoid biosynthetic pathway. The positive effect of light on the biosynthesis of caffeic acid derivatives has been observed in transformed roots of some other plant species, such as* Echinacea purpurea* [35] and* Cichorium intybus* [36].

5-*O*-caffeoylquinic acid (chlorogenic acid, compound** 2**) (Table 4, Figure 4) was the main constituent of the monocaffeoylquinic acid derivatives detected in the transformed roots of* R. carthamoides*. Its content ranged from 1.96 mg g^−1^ DW to 5.12 mg g^−1^ DW and was higher in HR-L root culture (Table 4). The amounts were much higher than chlorogenic acid level in transformed roots of* Echinacea purpurea* [35, 37],* Fagopyrum tataricum* [38], or* Polygonum multiflorum* [39]. In transformed roots of* R. carthamoides* the chlorogenic acid was further esterified with caffeic acid to produce the 3,5-di-*O*-caffeoylquinic acid (compound** 12**) (Table 4). The amount of the compound was 3.08 mg g^−1^ DW in HR-L and 1.92 mg g^−1^ DW in HR-D (Table 4). Additionally, in extracts of HR-L and HR-D four other dicaffeoylquinic acids were found but at considerably lower amounts compared with 3,5-*O*-dicaffeoylquinic acid (Table 4). In both types of* R. carthamoides* hairy root culture, the predominant fraction was tricaffeoylquinic acid derivatives (8.01 mg g^−1^ DW and 5.89 mg g^−1^ DW in HR-L and HR-D hairy roots, resp.) with compound** 16** (substituted tricaffeoylquinic acid) being the most abundant component. This compound represented up to 75% of the sum of the tricaffeoylquinic acids detected in transformed roots (Table 4). The tricaffeoylquinic acids and their derivatives are less common in plants than mono-, and dicaffeoyl ones [34]. This type of compounds was earlier identified in other species of family Asteraceae, such as* Arnica montana* [21] and* Erigeron breviscapus* [20]. To date there have been no reports on tricaffeoylquinic acid production in* R. carthamoides*.

Considerable differences in qualitative and quantitative profiles of phytochemicals between transformed roots and normal roots of soil-grown plants of* R. carthamoides* (SR) were observed. The production of caffeoylquinic acids and their derivatives was 2-3-times higher in SR roots than in transformed roots. The most prominent component of SR roots was chlorogenic acid (18.26 mg g^−1^ DW) (Table 4). The SR roots accumulated 17–23 times less of compound** 16** (tricaffeoylqunic acid derivative) than transformed roots, which was dominant component in the latter. Moreover, 1,4,5-tri-*O*-caffeoylquinic acid (compound** 15**) (Table 4) was identified only in the hairy root cultures. The differences between transformed roots and nontransformed roots of* R. carthamoides* were also observed in respect to other groups of secondary metabolites. Only hairy roots were able to produce the flavonoid glycosides (quercetagetin, quercetin, luteolin, and patuletin hexosides) when they were cultured in the light conditions. The total flavonoid content in this sample was 2.93 mg g^−1^ DW (Table 4). A comparative study of the ecdysteroids of transformed and normal roots of* R. carthamoides* showed that 20-hydroxyecdysone (compound** 8**) was only produced in the latter, reaching a level of 5.6 mg g^−1^ DW (Table 4).

The results of the present study showed that the transformation by* A. rhizogenes* strain A4 led to important modification of the metabolic pathways. Differences in the chemical profiles of transformed and normal roots have been also reported in other plant species [40, 41] which indicate that the insertion of Ri T-DNA interferes with the biosynthesis of the secondary metabolites. However, the differences observed between transformed and normal roots of* R. carthamoides* with regard to the qualitative and quantitative spectra of secondary metabolites could be also caused by differences in the developmental stage of roots or by environmental conditions (*in vitro* or* in vivo*).

## 4. Conclusions

The present study demonstrates that hairy roots of* R. carthamoides* are easily grown in liquid WPM medium. They produce a substantial biomass of approximately 90 g L^−1^ of fresh weight in 80 mL medium after a short cultivation period of 35 days. The establishment of hairy root culture with highly increased levels of tricaffeoylquinic acids and their derivatives observed in the present study indicates that the hairy roots can be used as potential sources of these secondary metabolites instead of the normal roots of soil-grown plants. This is especially important because tricaffeoylquinic acids have been shown to possess antimutagenic, antihyperglycemic, strong antioxidant, and radical scavenging effects. However, it has been found that the tricaffeoylquinic acids have more biological activity than mono- and dicaffeoylquinic acid derivatives [42–44]. The antioxidant activity of hairy roots is currently under investigation.

## Figures and Tables

**Table 1:** Induction of hairy roots of *R. carthamoides* derived from leaf explants by direct infection with *Agrobacterium rhizogenes* strains A4 and ATCC 15834.

Bacterial strain	Site of infection	Medium	% of explants forming roots	Mean number of roots/explants	Root length (cm)
A4	Petiole	MS	27.8^a^	3.0 ± 0.20^a^	1.6 ± 0.10^a^
		MS + AcS	37.3^b^	3.1 ± 0.14^a^	1.7 ± 0.08^a^
	Lamina	MS	35.0^ab^	2.1 ± 0.12^b^	1.5 ± 0.10^a^
		MS + AcS	43.3^b^	2.1 ± 0.09^b^	1.6 ± 0.08^a^

ATCC 15834	Petiole	MS	17.3^cd^	1.7 ± 0.22^bc^	1.2 ± 0.13^a^
		MS + AcS	22.7^d^	1.3 ± 0.11^c^	1.2 ± 0.11^a^
	Lamina	MS	13.3^c^	1.5 ± 0.19^c^	1.3 ± 0.11^a^
		MS + AcS	18.3^cd^	1.3 ± 0.12^c^	1.6 ± 0.14^a^

Explants were infected with a needle dipped in the bacterial culture and placed on hormone-free MS agar (0.7%) medium without (MS) or with 200 *μ*M acetosyringone (MS + AcS). Observations were recorded after 5 weeks. Each treatment involved 25–35 explants. The experiments were repeated three times. Values are given as the mean ± SE (standard error). The experiment means followed by the same superscript letter within the column are not significantly different at the level of *P* ≥ 0.05 (the Mann-Whitney *U* test).

**Table 2:** Specific pairs of primers used for PCR analysis of transformed roots of *R. carthamoides*.

Gene	Primer sequences	Amplified fragments (bp)
*rol*A	5′ CTA AGG TCA AGA AGA AGA AGG 3′ 5′ AGA AGT TAT AGC CAG AGG AGT 3′	107

*rol*B	5′ GCT CTT GCA GTG CTA GAT TT 3′ 5′ GAA GGT GCA AGC TAC CTC TC 3′	386

*rol*C	5′ CTC CTG ACA TCA AAC TCG TC 3′ 5′ TGC TTC GAG TTA TGG GTA CA 3′	582

*rol*D	5′ GAT GAT TTT CGT TTT ATC AAG 3′ 5′ GAA GGA CAG AGG ATA CTT AAA C 3′	204

*aux*1	5′ ATC TTA GTC ACT TCA TAG CAG TT 3′ 5′ CTT TTT GAG ATA GAA GAA CAA G 3′	500

*aux*2	5′ ATA TCT GCT TCA ACA AAA GTA AC 3′ 5′ TGA GTT AAT CGA AAC GAT AAT A 3′	774

*vir*G	5′ AGT TCA ATC GTG TAC TTT CCT 3′ 5′ CTG ATA TTC AGT GTC CAG TCT 3′	319

**Table 3:** UPLC-PDA-ESI-MS^3^ data of detected and identified polyphenols in hairy roots and in the roots of soil-grown plant extracts of *R. carthamoides*.

Number	Compound	*t*_*R*_ (min)	UV (nm)	[M–H]^−^ *m*/*z*	MS^2^ ions	MS^3^ ions
**1**	3-*O*-Caffeoylquinic acid	6.6	294sh, 325	353	191^b^, 179, 135	—
**2**	5-*O*-Caffeoylquinic acid^a^	10.9	294sh, 325	353	191^**b**^, 179, 135	171^b^, 127, 85^b^
**3**	4-*O*-Caffeoylquinic acid	12.9	294sh, 325	353	191, 179, 173^b^	—
**4**	1,3-Di-*O*-caffeoylquinic acid^a^	19.4	294sh, 328	515	353^**b**^, 335, 191, 179	191^b^, 179, 135
**5**	Quercetagetin hexoside	22.4	259, 356	479	385, 317^b^	—
**6**	Quercetin hexoside	25.1	253, 365	463	301^**b**^	179^b^, 151
**7**	Quercetin hexoside	26.5	254, 368	463	301^b^	179^b^, 151
**8**	20-Hydroxyecdysone^a^	27.4	247	479, 525^c^	479^b^	—
**9**	Luteolin hexoside	28.0	255, 349	447	285^**b**^	241^b^, 223, 213, 175, 151
**10**	Patuletin hexoside	28.6	254, 365	493	331^**b**^, 316	316^b^, 287
**11**	3,4-Di-*O*-caffeoylquinic acid	31.9	294sh, 325	515	353^**b**^, 335, 317, 299, 203	191, 179, 173^b^
**12**	3,5-Di-*O*-caffeoylquinic acid	32.5	294sh, 328	515	353^**b**^, 191	191^b^, 179
**13**	1,5-Di-*O*-caffeoylquinic acid	33.0	294sh, 328	515	353^**b**^, 191	191^b^, 179
**14**	4,5-Di-*O*-caffeoylquinic acid	36.2	294sh, 328	515	353^**b**^, 335, 299, 255, 203, 173	191, 179, 173^b^
**15**	1,4,5-Tri-*O*-caffeoylquinic acid	40.4	294sh, 325	677	515^b^, **497**, 353, 335	353^b^, 335, 191, 179
**16**	Tricaffeoylquinic acid derivative	43.0	294sh, 327	793	631^**b**^, 613, 515, 498, 469	515, 469^b^, 353
**17**	Tricaffeoylquinic acid derivative	45.2	294sh, 329	793	631^**b**^, 469, 353	469^b^, 353

^a^Identified by the reference standard.

^b^Base peak. Bold-ions were subjected to MS^3^fragmentation.

^c^[M + HCOO]^−^.

**Table 4:** Results of HPLC-PDA quantification of polyphenols and 20-hydroxyecdysone in hairy roots and in the roots of soil-grown plant extracts of *R*. *carthamoides*.

Number	Compound	*t*_*R*_ (min)	HR-L	HR-D	SR
			mg g^−1^ DW	mg g^−1^ DW	mg g^−1^ DW
**1**	3-*O*-Caffeoylquinic acid	4.0	0.06 (4.80)^a^	—	0.70 (0.34)^b^
**2**	5-*O*-Caffeoylquinic acid	7.4	5.12 (2.24)^b^	1.96 (2.92)^a^	18.26 (0.46)^c^
**3**	4-*O*-Caffeoylquinic acid	8.1	0.12 (5.03)^b^	0.06 (2.35)^a^	0.42 (0.95)^c^
**4**	1,3-Di-*O*-caffeoylquinic acid	10.2	0.22 (3.90)^b^	0.12 (2.68)^a^	1.21 (1.88)^c^
**5**	Quercetagetin hexoside	10.7	0.96 (1.65)	—	—
**6**	Quercetin hexoside	11.4	0.93 (1.71)	—	—
**7**	Quercetin hexoside	11.9	0.52 (0.61)	—	—
**8**	20-Hydroxyecdysone	12.0	—	—	5.60 (1.36)
**9**	Luteolin hexoside	12.1	0.27 (1.43)	—	—
**10**	Patuletin hexoside	12.2	0.25 (1.24)	—	—
**11**	3,4-Di-*O*-caffeoylquinic acid	13.1	0.22 (0.95)^b^	0.15 (3.67)^a^	0.52 (2.62)^c^
**12**	3,5-Di-*O*-caffeoylquinic acid	13.4	3.08 (3.32)^b^	1.92 (2.75)^a^	8.47 (0.51)^c^
**13**	1,5-Di-*O*-caffeoylquinic acid	13.9	0.28 (3.28)^b^	0.12 (3.01)^a^	1.44 (2.75)^c^
**14**	4,5-Di-*O*-caffeoylquinic acid	14.1	1.97 (3.39)^c^	1.23 (1.77)^b^	1.04 (2.05)^a^
**15**	1,4,5-Tri-*O*-caffeoylquinic acid	15.3	1.38 (4.67)^b^	1.08 (2.86)^a^	—
**16**	Tricaffeoylquinic acid derivative	15.6	5.97 (4.88)^c^	4.34 (2.09)^b^	0.26 (2.18)^a^
**17**	Tricaffeoylquinic acid derivative	16.4	0.66 (0.24)^b^	0.47 (4.58)^a^	2.88 (1.38)^c^

Results are mean values of triplicate analyses calculated per DW of the plant material; the values in parentheses are relative standard deviations RSD (%); different superscript letter within the rows indicates significant differences in the mean values at *P* < 0.01 (one-way ANOVA by Tukey's test).

HR-L: hairy roots cultured in the WPM liquid medium, in the presence of light.

HR-D: hairy roots cultured in the WPM liquid medium, in darkness.

SR: roots of 3-year-old soil-grown plants.
